# Immune response after experimental allergic encephalomyelitis in rats subjected to calorie restriction

**DOI:** 10.1186/1742-2094-4-6

**Published:** 2007-01-25

**Authors:** Ana I Esquifino, Pilar Cano, Vanessa Jimenez-Ortega, María P Fernández-Mateos, Daniel P Cardinali

**Affiliations:** 1 Departamento de Bioquímica y Biología Molecular III, Facultad de Medicina, Universidad Complutense, 28040 Madrid, Spain; 2 Departamento de Biología Celular, Facultad de Medicina, Universidad Complutense, 28040 Madrid, Spain; 3 Departamento de Fisiología, Facultad de Medicina, Universidad de Buenos Aires, 1121 Buenos Aires, Argentina

## Abstract

Male Lewis rats (6 weeks-old) were submitted to a calorie restriction equivalent to 33% or 66% of food restriction. Fifteen days later, groups of 7 animals were injected with complete Freund's adjuvant plus spinal cord homogenate (SCH) to induce experimental allergic encephalomyelitis (EAE) or with complete Freund's adjuvant alone. EAE was defined solely on clinical grounds. Rats were assessed daily for clinical signs of EAE and were killed on day 15 after immunization. Both diet and SCH injection diminished body weight significantly. In contrast to rats receiving a normal diet or a 33% calorie-restricted diet, rats subjected to severe calorie restriction did not exhibit clinical signs of EAE. Concomitantly with the lack of disease manifestation, 66% of calorie-restricted rats injected with SCH showed significantly less splenic and lymph node mitogenic response to concanavalin A (Con A) and a higher splenic response to lipopolysaccharide. Fewer splenic, lymph node and thymic CD4^+ ^cells, greater numbers of splenic and lymph node CD8^+ ^and CD4^+^- CD8^+ ^cells, and fewer splenic T, B and T-B cells, and lymph node and thymic B and T-B cells were observed. There was impaired interferon (IFN)-γ production occurred in the three examined tissues. The results are compatible with the view that the acute phase of EAE can be curtailed by severe calorie restriction, presumably through impaired IFN-γ production.

## Background

Multiple sclerosis (MS) is an autoimmune disease that results in demyelination of axonal tracts in the CNS causing a wide range of neurological symptoms [1]. Major histocompatibility (MHC) class II-restricted CD4^+ ^T-cells that recognize CNS components are the predominant pathogenic mediators in MS and act by secreting inflammatory cytokines such as IFN-γ. Epidemiological studies suggest that unidentified environmental factors contribute to the etiology of MS [2,3] and diet is a commonly postulated factor because strong associations have been observed between increased MS prevalence and diets high in meat and dairy products and low in fish [1,4-6].

These epidemiological findings have provided a rationale for a number of clinical trials aimed to establish beneficial effect of dietary interventions in MS, with heterogeneous results [1]. There have been reports indicating that a diet with a very low saturated fat content may provide long term benefits for rates of mortality, relapse severity and disability in MS, particularly if initiated during the earliest stages of the disease [7,8].

While the nature of the event(s) in MS that lead to activation and proliferation of T-cells is unknown, a similar disease can be induced in rodents by subcutaneous (s.c.) injection of either spinal cord homogenate (SCH) or CNS antigens including myelin basic protein, myelin oligodendrocyte glycoprotein, or proteolipid protein; or phenotypic peptides of these [9]. Besides different triggering mechanisms, experimental allergic encephalomyelitis (EAE) animal models share many characteristics of MS [10]. This includes an "activation phase" where antigen-presenting cells process the immunized antigen, migrate to the lymph nodes and present immunodominant peptides to naïve T-cells; and an "effector phase" where CD4^+ ^T-cells that recognize antigen proliferate and cross the blood-brain barrier to lead an inflammatory attack that results in demyelinated lesions. In most models, the T helper 1 (Th1) subset of T-cells has been implicated in the induction of EAE.

In a previous study [11] we reported the inhibitory effect of a severe caloric restriction (i.e., a 66 % reduction of calorie intake) on the development of EAE in Lewis rats. Calorie-restricted rats did not exhibit the augmented lymph node mitogenic response to concanavalin A (Con A) following SCH immunization found in controls, nor the increase in plasma ACTH and corticosterone found after SCH immunization [11].

The present study was carried out to further examine the immune responses after EAE in rats subjected to a severe (i.e., 66 %) or a moderate (i.e. 33 %) calorie restriction. The mitogenic responses and lymphocyte subset groups of spleen, submaxillary lymph node (SmLN) and thymus were assessed. The changes in immune parameters were correlated with the release of interferon (IFN)-γ in vitro by immunocompetent cells.

## Materials and methods

Male Lewis rats (6 weeks old, 140–170 g) were purchased from Charles River S.A., Spain, and were individually housed in a standard animal facility. Rats were put in individual cages to avoid cannibalism among calorie restricted animals [12]. Control rats (n = 14) had free access to an equilibrated diet (AIN-93G, Diets Inc., Pennsylvania, USA),) and water for 4 weeks. Severely calorie-restricted rats (n = 14) had daily access to 7 g of an unbalanced AIN-93G diet enriched in proteins and low in fat and carbohydrates [13] and water *ad libitum *for 4 weeks. This calorie restriction was equivalent to a 66% food restriction. A second group of 14 rats had daily access to 14 g of an unbalanced AIN-93G diet enriched in proteins and low in fat and carbohydrates (33% calorie restriction). The experiments were conducted in accordance with the guidelines of the International Council for Laboratory Animal Science (ICLAS). Protocols were approved by the Institutional Animals Ethics Committee. Spinal cord obtained from adult Wistar rats was homogenized in PBS buffer at a concentration of 1 g/mL to serve as an immunogen.

After 15 days of calorie restriction, each group of rats (control and moderate or severely calorie-restricted rats) were divided in two subgroups as follows: a) animals receiving complete Freund's adjuvant (n = 7); b) animals receiving complete Freund's adjuvant plus SCH (n = 7). Rats were immunized by the s.c. injection of a mixture of SCH and complete Freund's adjuvant containing Mycobacterium tuberculosis H37Ra (5 mg/mL; Difco Laboratories, Detroit, Michigan) (v/v) in a final volume of 200 μl. Animals were assessed daily for clinical signs of EAE using the following criteria: 0, normal; 0.5, loss of tonicity in distal half of tail; 1, piloerection; 2, total loss of tail tonicity; 3, hind leg paralysis; 4, paraplegia; and 5, moribund.

Animals were killed by decapitation on day 15 after immunization (7 animals per group) and blood was collected from the trunk wound in heparinized tubes and was centrifuged at 1500 × g for 15 min. The spleen, SmLN and thymus nodes were removed aseptically, weighed and placed in Petri disks containing balanced salt solution; the cells were then gently teased apart. After removing the clumps by centrifugation, the cells were suspended in sterile supplemented medium (RPMI 1640), containing 10% heat-inactivated, fetal bovine serum, 20 mM L-glutamine, 0.02 mM 2-mercaptoethanol and gentamicin (50 mg/ml), and were counted.

Mitogen assays were performed as described in detail elsewhere [14]. Splenic, SmLN or thymic cells were used at a final number of 5 × 10^4^cells per 0.1-ml well. Control and experimental cultures were run in triplicate. Mitogens were added to the cultures at final supramaximal concentrations of 5 μg/ml. The cultures were incubated in a humidified 37°C incubator in an atmosphere of 5% CO_2_. After a 48 h incubation, ^3^H-thymidine (0.2 μCi) was added to each well in a volume of 0.02 ml. Cells were harvested 5 h later using an automated sample harvester, and the filters were counted in a liquid scintillation spectrometer. The proliferation index was estimated as the ratio between cells stimulated in the presence of mitogens and controls. Results were expressed as proliferation index/number of cells.

The relative size distributions of lymph cells in spleen, SmLN and thymus were determined by FACS analysis, as previously described [15]. For these studies, we used the following monoclonal antibodies: Anti-rat LCA (OX-33) for B lymphocytes (Serotec, Oxford, UK), anti-rat TCR alpha/beta (R7.3) for T lymphocytes (Serotec, Oxford, UK), anti-rat CD4 (OX-35) which recognize a rat T helper cell differentiation antigen (Pharmingen, San Diego, CA, USA), and anti-rat CD8a (OX-8) which recognize the reactive antigen expressed on rat T cytotoxic/suppressor cells (Pharmingen, San Diego, CA, USA). Lymphocytes, isolated as indicated above, were washed in cold PBS with 0.02% sodium azide and then incubated (3 × 10^5 ^cells/tube) with appropriate primary antibodies for 30 min at 4°C. Following two washes, the cells were incubated with 1 ml of PBS-BSA 1%, during 5 min at 4°C, washed three times, resuspended in 1% paraformaldehyde in PBS. Fluorescence intensity was analyzed by fluorescence-activated cell sorting (FACStarplus; Beckton Dickinson, Mountain View, CA). Dead cells were excluded by gating with propidium iodide.

For analyzing IFN-γ release, splenic, SmLN or thymic cells (10^5^/100 μl) were incubated for 24 h, their media removed and, after adding fresh media including all components, they were incubated for 24 h more. Both media were collected and pooled for IFN-γ measurement. The incubations were performed in triplicates. Microscopical examination of the cell preparations used indicated that > 95% were lymph node cells. Neither treatment affected the viability of the cells. IFN-γ concentration in media culture was measured after centrifugation to remove adherent cells. An ELISA commercial kit from Endogen (Woburn, MA, USA), previously validated in our laboratory, was employed [16]. The assay was performed as follows: 100 μl of standards or unknown samples were added to each antibody-coated well, and the plates were incubated overnight at room temperature. The reaction was stopped by washing thrice with wash buffer (2% Tween 20 in 50 mM Tris, pH 3.6). The wells were incubated with 100 μl of biotynilated detecting antibody at the titter previously tested. After 1 h at room temperature the reaction was stopped by washing thrice with wash buffer. One hundred μl of streptavidin-HRP solution (in Dulbecco's phosphate-buffered saline, pH 7.4) was then added and the samples were incubated for 30 min. The reaction was stopped by adding 100 μl of 0.18 M sulfuric acid. The plates were read within 30 min in an ELISA reader set at 450 nm and 550 nm. Values were obtained by subtracting the reading at 550 nm from the reading at 450 nm, to correct for any optical defect of microtiter plate. IFN-γ release was expressed as pg/mL/48 h incubation. Sensitivity of the assay was 100 pg/mL.

Statistical analysis of results was performed by a two-way factorial analysis of variance (ANOVA) and a one-way ANOVA followed by a Bonferroni's test. P values lower than 0.05 were considered evidence for statistical significance.

## Results

**Figure 1:**
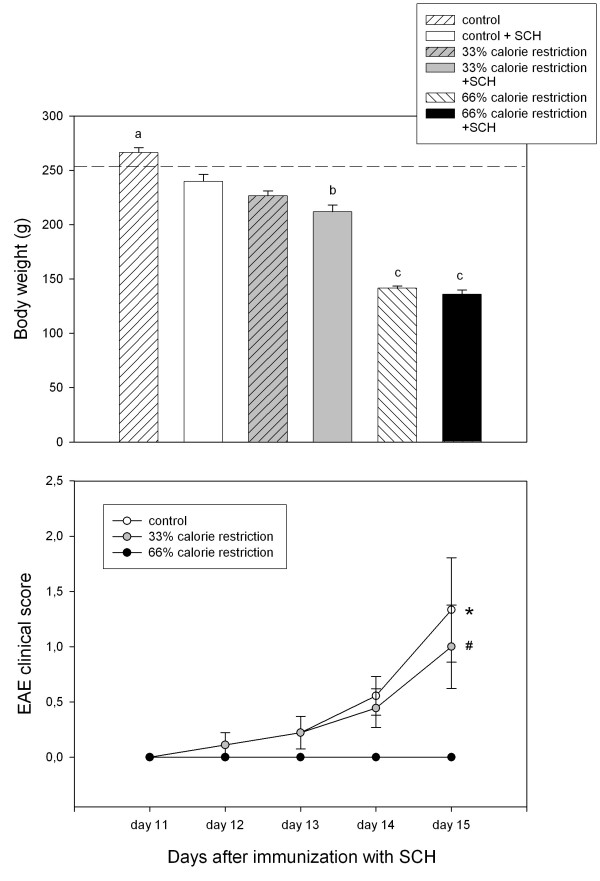
Body weight at sacrifice (upper panel) and clinical evolution of EAE in control and 33% and 66% calorie-restricted rats (lower panel). Male Lewis rats were kept for 1 month under a control or restricted diet, as described in Materials and methods. On day 15 the rats received complete Freund's adjuvant plus spinal cord homogenate (SCH) or complete Freund's adjuvant alone. Rats were assessed daily for clinical signs of EAE using the clinical criteria described in Materials and methods. Data shown as mean ± SEM. Upper panel: numbers designate the groups whose means differed significantly in a one-way ANOVA followed by a Bonferroni's test, as follows: ^a ^p < 0.02 vs. all groups; ^b ^p < 0.03 vs. control rats injected with SCH. ^c ^p < 0.01 vs. control and 33% calorie-restricted rats. Dotted line: average weight of intact rats. Lower panel: * p < 0.05 vs. all time points; # p < 0.05 vs. day 12 or day 13, one-way ANOVA followed by a Bonferroni's test. For further statistical analysis, see text.

Figure 1 shows final body weight (upper panel) and the evolution of the clinical scores of EAE in control and calorie restricted rats (lower panel). When analyzed as main factors in a factorial ANOVA, both calorie restriction and SCH injection decreased body weight significantly (F_2,36 _= 294, p < 0.00001 and F_1,36 _= 15.7, p = 0.0003).

Rats subjected to the normal diet or to a 33% calorie restriction exhibited clinical signs of the disease, starting on day 12 after SCH injection whereas, a 66%-calorie restriction effectively suppressed the course of EAE in Lewis rats (Fig. 1, lower panel). Rats receiving complete Freund's adjuvant alone and subjected to none, 33% or 66% calorie restriction did not exhibit any sign of disease (results not shown).

**Figure 2:**
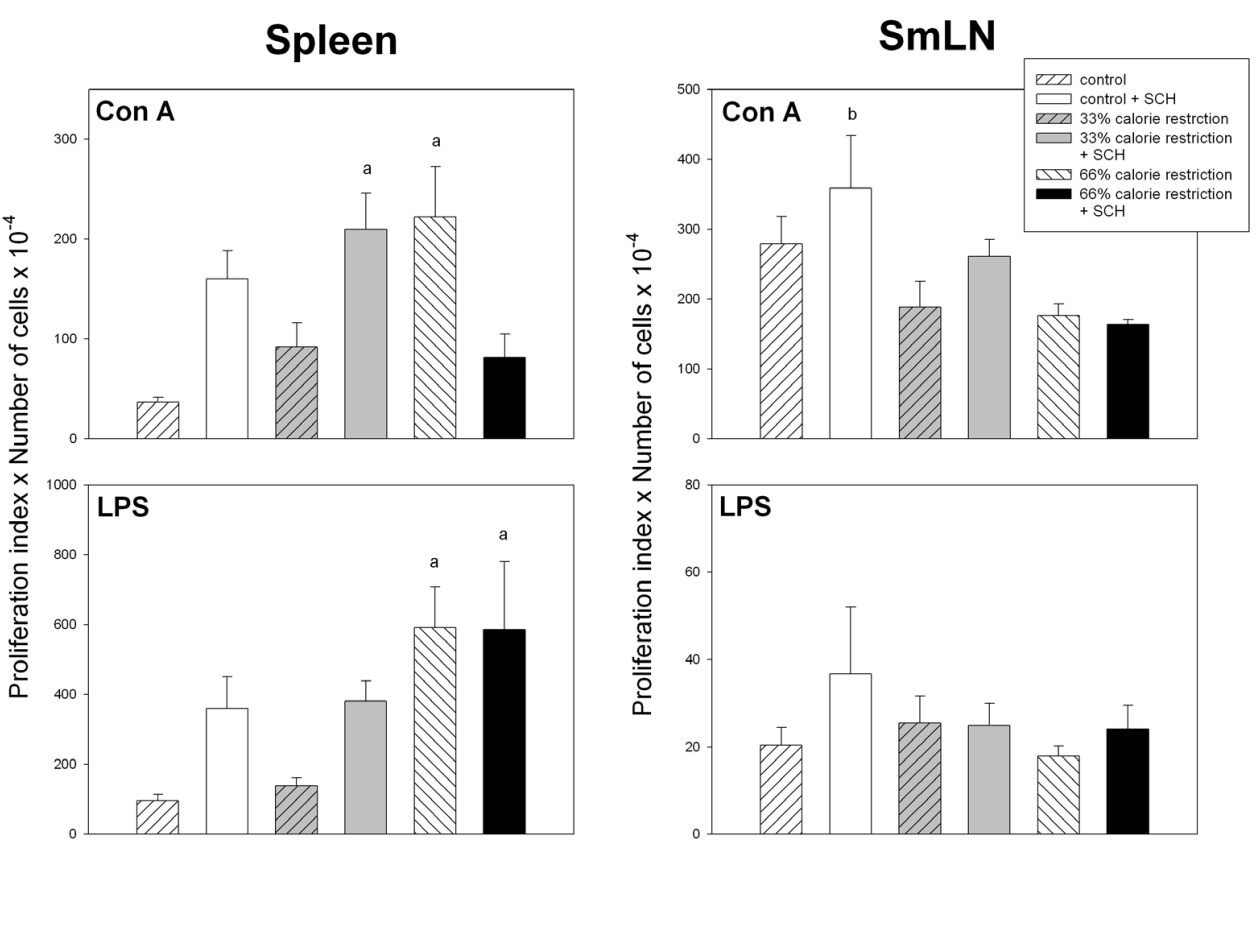
Mitogenic responses to Con A and LPS in spleen (left panels) and SmLN (right panels) of control and calorie-restricted rats. Data shown as mean ± SEM (n = 7 rats/group). Letters indicate the existence of significant differences between groups after a one-way ANOVA followed by a Bonferroni's test, as follows: ^a ^p < 0.02 vs. control rats not injected with SCH; ^b ^p < 0.03 vs. 66% calorie-restricted rats. For further statistical analysis, see text.

Figure 2 depicts the mitogenic responses to Con A and LPS of cells derived from spleen or SmLN of control or calorie-restricted rats. In the case of splenic Con A mitogenic response, a significant interaction "diet x immunization" was found in factorial ANOVA (F_2,36 _= 3.48, p = 0.0415), i.e., SCH injection augmented Con A response in control and 33% calorie-restricted rats while decreased it under severe caloric restriction (Fig 2, upper left panel). Splenic cell response to LPS was higher in 66% calorie-restricted rats (F_2,36 _= 4.2, p = 0.0229, factorial ANOVA, Fig. 2, lower left panel).

In SmLN, a significant depression of Con A mitogenic activity was observed as a function of calorie restriction (F_2,36 _= 7.1, p = 0.0025, factorial ANOVA, Fig. 2, upper right panel). The changes in lymph node LPS mitogenic response (Fig. 2) or of mitogenic responses in thymic cells were not significant (results not shown).

**Figure 3:**
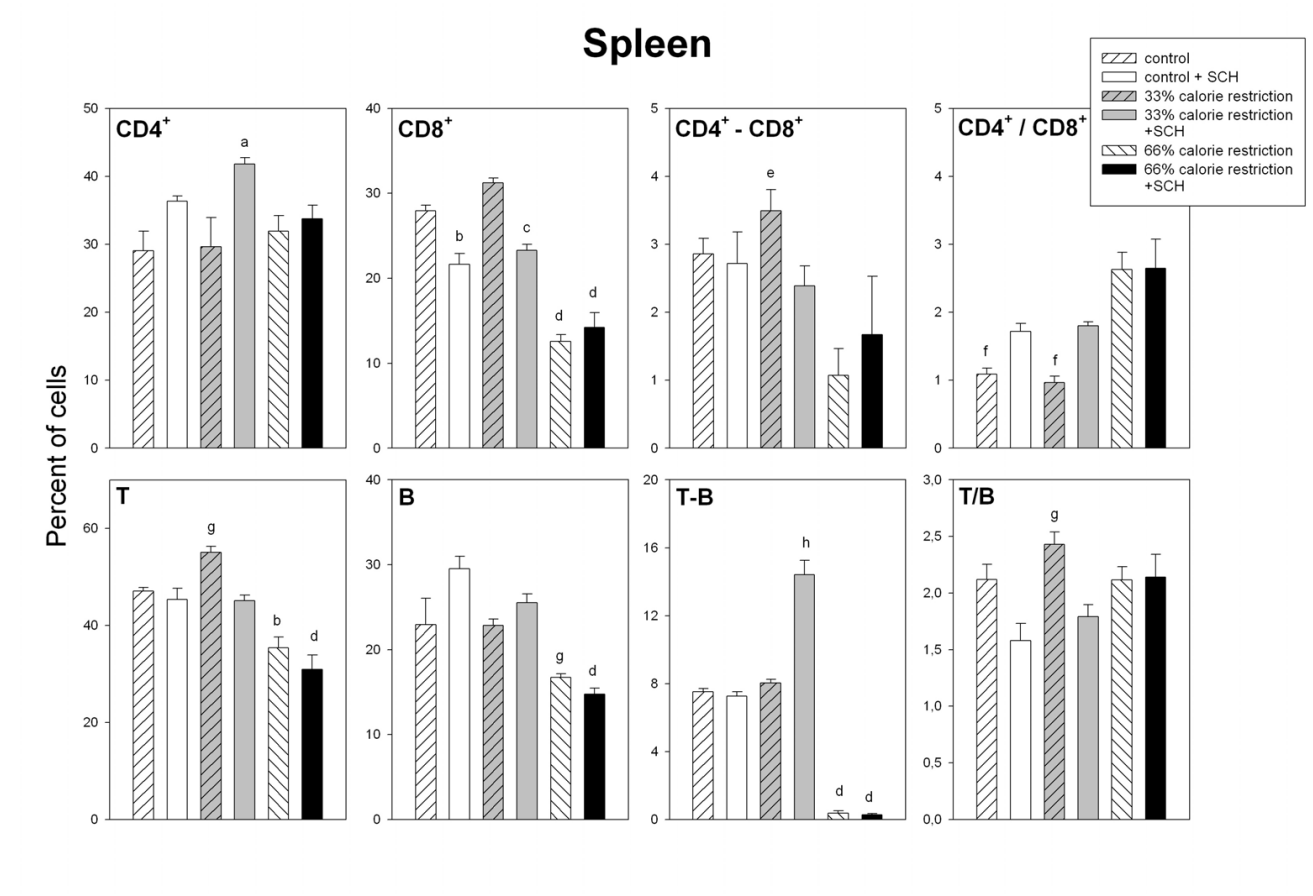
Relative size distributions of lymph cells in the spleen of control and calorie-restricted rats. CD4^+^, CD8^+^, CD4^+^-CD8^+^, T, B and T-B lymphocytes, as well as CD4^+^/CD8^+ ^and T/B ratios, were measured as described in Materials and methods. Data shown as mean ± SEM (n = 7 rats/group). Letters indicate the existence of significant differences between groups after a one-way ANOVA followed by a Bonferroni's test, as follows: ^a ^p < 0.05 vs. control and 33% calorie-restricted rats, neither injected with SCH; ^b ^p < 0.02 vs. control and 33% calorie-restricted rats, neither injected with SCH; ^c ^p < 0.02 vs. 33% calorie-restricted rats not injected with SCH; ^d ^p < 0.02 vs. control and 33% calorie-restricted rats regardless of SCH injection; ^e ^p < 0.02 vs. 66% calorie-restricted rats not injected with SCH; ^f ^p < 0.01 vs. 66% calorie-restricted rats regardless of SCH injection; ^g ^p < 0.03 vs. control and 33% calorie-restricted rats, both injected with SCH; ^h ^p < 0.01 vs. all groups. For further statistical analysis, see text.

**Figure 4:**
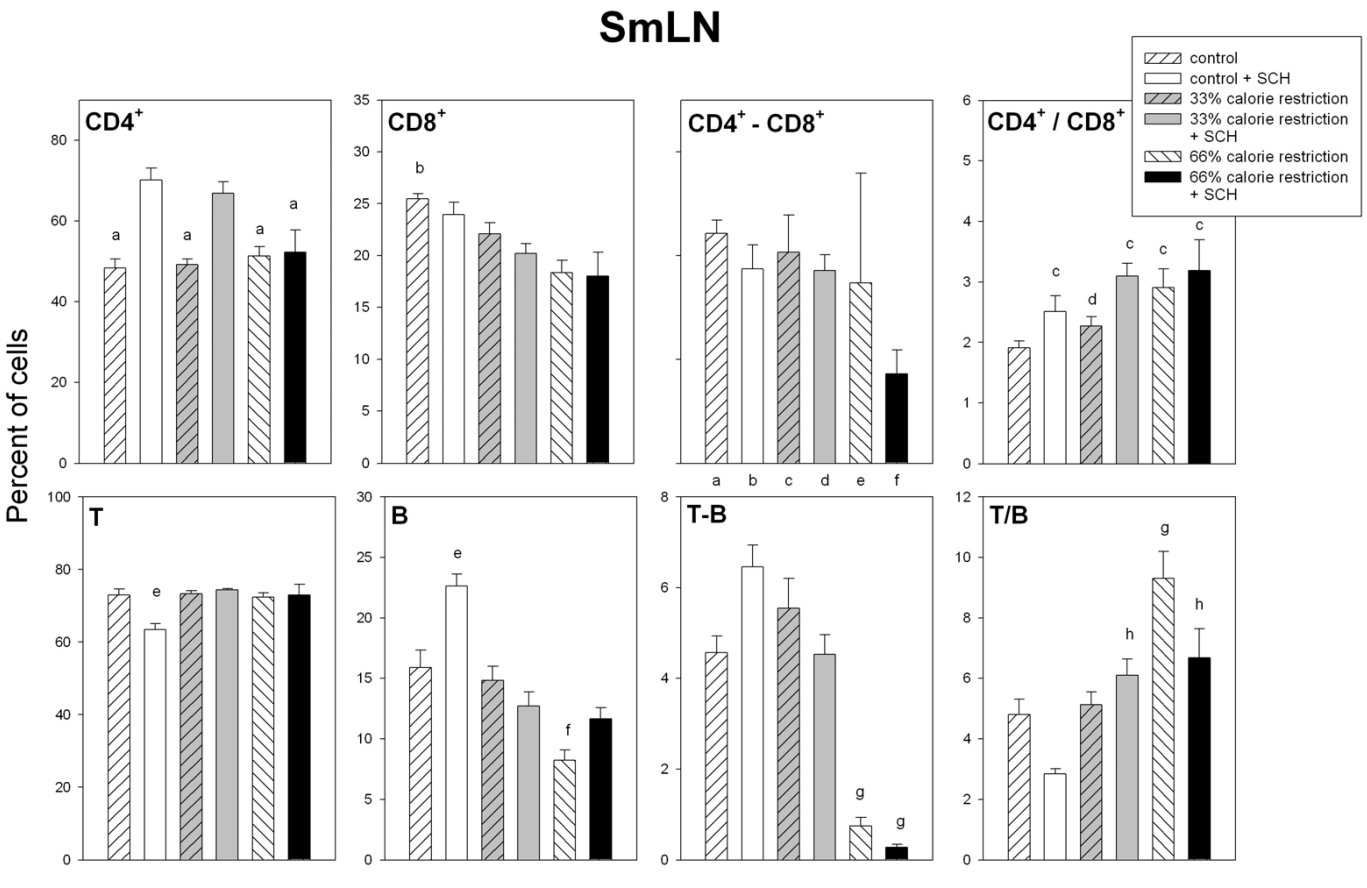
Relative size distributions of lymph cells in SmLN of control and calorie-restricted rats. CD4^+^, CD8^+^, CD4^+^-CD8^+^, T, B and T-B lymphocytes, as well as CD4^+^/CD8^+ ^and T/B ratios, were measured as described in Materials and methods. Data shown as mean ± SEM (n = 7 rats/group). Letters indicate the existence of significant differences between groups after a one-way ANOVA followed by a Bonferroni's test, as follows: ^a ^p < 0.02 vs. control and 33% calorie-restricted rats, both injected with SCH; ^b ^p < 0.02 vs. 66% calorie-restricted rats regardless of SCH injection; ^c ^p < 0.05 vs. control rats not injected with SCH; ^d ^p < 0.05 vs. 33% and 66% calorie-restricted rats, both injected with SCH; ^e ^p < 0.04 vs. all groups; ^f ^p < 0.02 vs. control rats injected or not injected with SCH and 33% calorie-restricted rats not injected with SCH; ^g ^p < 0.01 vs. control and 33% calorie-restricted rats regardless of SCH injection; ^h ^p < 0.01 vs. control rats injected with SCH. For further statistical analysis, see text.

**Figure 5:**
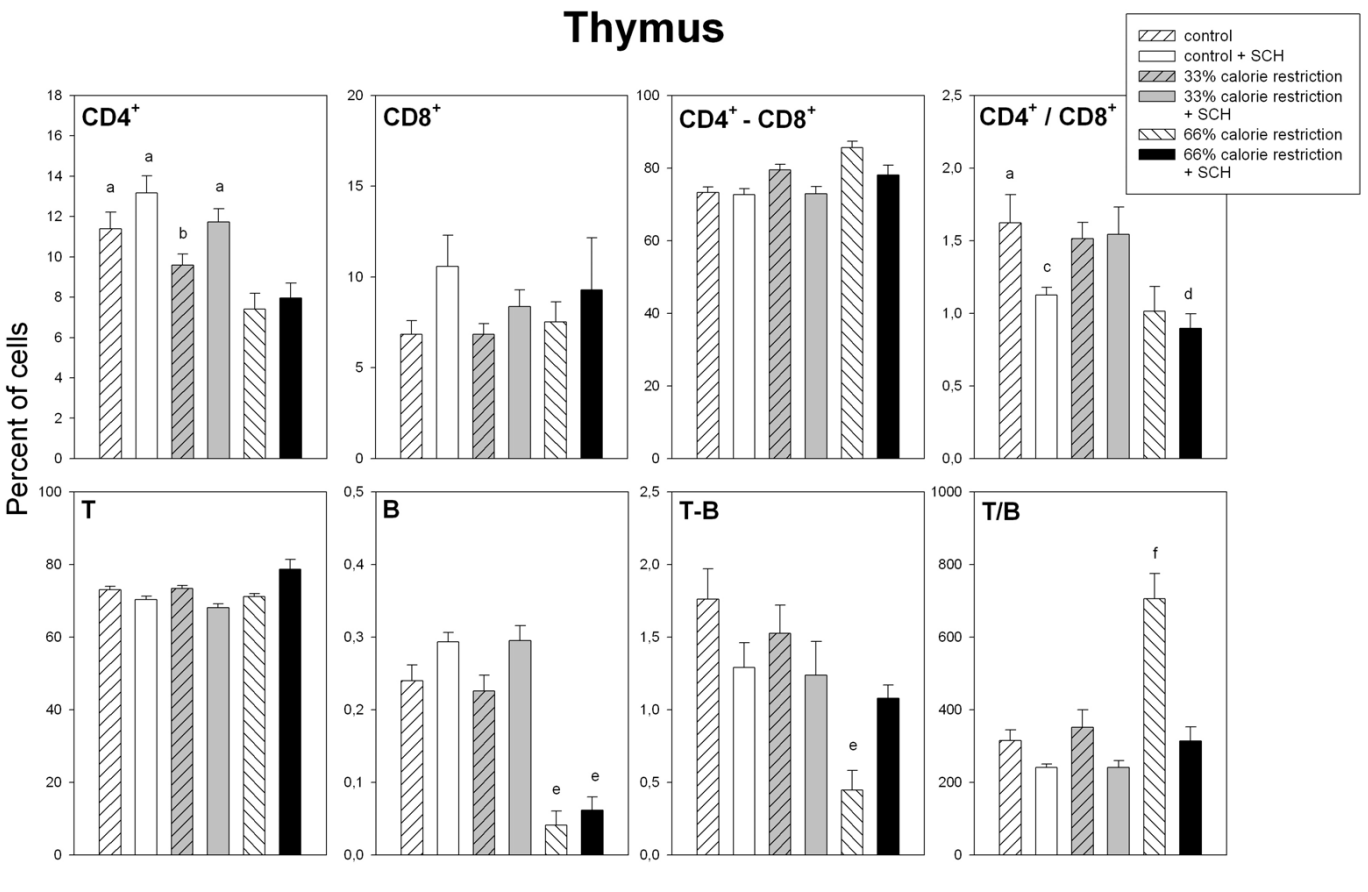
Relative size distributions of lymph cells in the thymus of control and calorie restricted rats. CD4^+^, CD8^+^, CD4^+^-CD8^+^, T, B and T-B lymphocytes, as well as CD4^+^/CD8^+ ^and T/B ratios, were measured as described in Materials and methods. Data shown as mean ± SEM (n = 7 rats/group). Letters indicate the existence of significant differences between groups after a one-way ANOVA followed by a Bonferroni's test, as follows: ^a ^p < 0.03 vs. 66% calorie-restricted rats regardless of SCH injection; ^b ^p < 0.03 vs. control rats injected with SCH; ^c ^p < 0.01 vs. control rats not injected with SCH; ^d ^p < 0.04 vs. 33% calorie-restricted rats regardless of SCH injection; ^e ^p < 0.01 vs. control and 33% calorie-restricted rats regardless of SCH injection; ^f ^p < 0.01 vs. all groups. For further statistical analysis, see text.

Figures 3 to 5 summarize the data on the different immune cell populations in spleen, SmLN and thymus of control and calorie restricted rats. A significant stimulatory effect of SCH immunization on splenic, lymph node and thymic CD4^+ ^cell number was found (F_1,36 _= 11.6, p = 0.0016; F_1,36 _= 27.1, p < 0.001 and F_1,36 _= 18.8, p < 0.001, respectively, Fig. 3 to 5). A significant interaction "diet x immunization" was detected in the case of spleen and SmLN, SCH injection augmenting CD4^+ ^cell number in control and moderately calorie-restricted, but not in severely calorie-restricted animals (F_2,36 _= 3.44. p = 0.0429 and F_2,36 _= 6.17, p = 0.005, for spleen and SmLN, respectively, factorial ANOVA, Fig. 3 and 4).

Calorie restriction depressed splenic and lymph node CD8^+ ^cell number (F_2,36 _= 98.2, p < 0.0001 and F_2,36 _= 11.7, p = 0.0001, Fig. 3 and 4), with a significant interaction "diet x immunization" in the case of spleen (F_2,36 _= 11.9, p = 0.0001), i.e., the decrease in CD8^+ ^cell number observed after SCH immunization in control and 33% calorie restricted rats was no longer found in 66% calorie-restricted animals (Fig. 3). Thymic CD8^+ ^cells augmented after SCH immunization (F_1,36 _= 10.2, p = 0.0029, factorial ANOVA, Fig. 5). Consequently, CD4^+^/CD8^+ ^ratios increased after calorie restriction or SCH immunization in spleen (F_2,36 _= 20.8, p < 0.0001 and F_1,36 _= 7.53, p = 0.0094) and SmLN (F_2,36 _= 4.77. p = 0.0145 and F_1,36 _= 26.5, p < 0.0001) (Fig. 3 and 4) and decreased after calorie restriction in thymus (F_2,36 _= 9.02, p = 0.0007, Fig. 5). Splenic double-labeled CD4^+^-CD8^+ ^cells decreased after calorie restriction (F_2,36 _= 6.99, p = 0.0027, factorial ANOVA, Fig. 3).

In the spleen, T cell number decreased as a function of calorie restriction and augmented after SCH immunization (F_2,36 _= 43, p < 0.0001 and F_1,36 _= 11.8, p = 0.0015, factorial ANOVA, Fig. 3). The decrease of lymph node T cells seen in control following SCH immunization was not longer observed after a moderate or a severe calorie restriction (F_2,36 _= 5.91, p = 0.006 for the interaction "diet x immunization" in the factorial ANOVA, Fig. 4). Likewise, a significant interaction "diet x immunization" occurred for thymic T cell number, i.e., in contrast to the decrease after immunization seen in control and 33% calorie-restricted rats there was an increase in 66% calorie-restricted rats (F_2,36 _= 9.91, p = 0.0004 for the interaction "diet x immunization" in the factorial ANOVA, Fig. 5).

Splenic, lymph node and thymic B cell number decreased after calorie restriction (F_2,36 _= 22.3, 26.8 and 88, p < 0.0001) (Fig. 3 to 5). In the case of spleen and SmLN, significant interactions "diet x immunization" occurred (F_2,36 _= 3.39, p = 0.0448 and F_2,36 _= 8.16, p = 0.0012, respectively), the stimulatory effect of SCH immunization seen in controls being no longer observed in 33% or 66% calorie-deprived rats (Fig. 3 and 4). As a consequence, a significant interaction between diet and immunization occurred for T/B ratio in every tissue, i.e., the decrease after SCH injection taking place in controls disappeared or was reversed in calorie-restricted rats. Double labeled T-B cells in spleen and lymph nodes decreased markedly in severely calorie-restricted rats (p < 0.0001, Fig. 3 and 4). A significant interaction "diet x immunization" was observed in the thymus (F_2,36 _= 5.81, p = 0.0065), T-B cells augmenting in 66% calorie restricted rats, but not in control or 33% calorie-restricted animals (Fig. 5).

**Figure 6:**
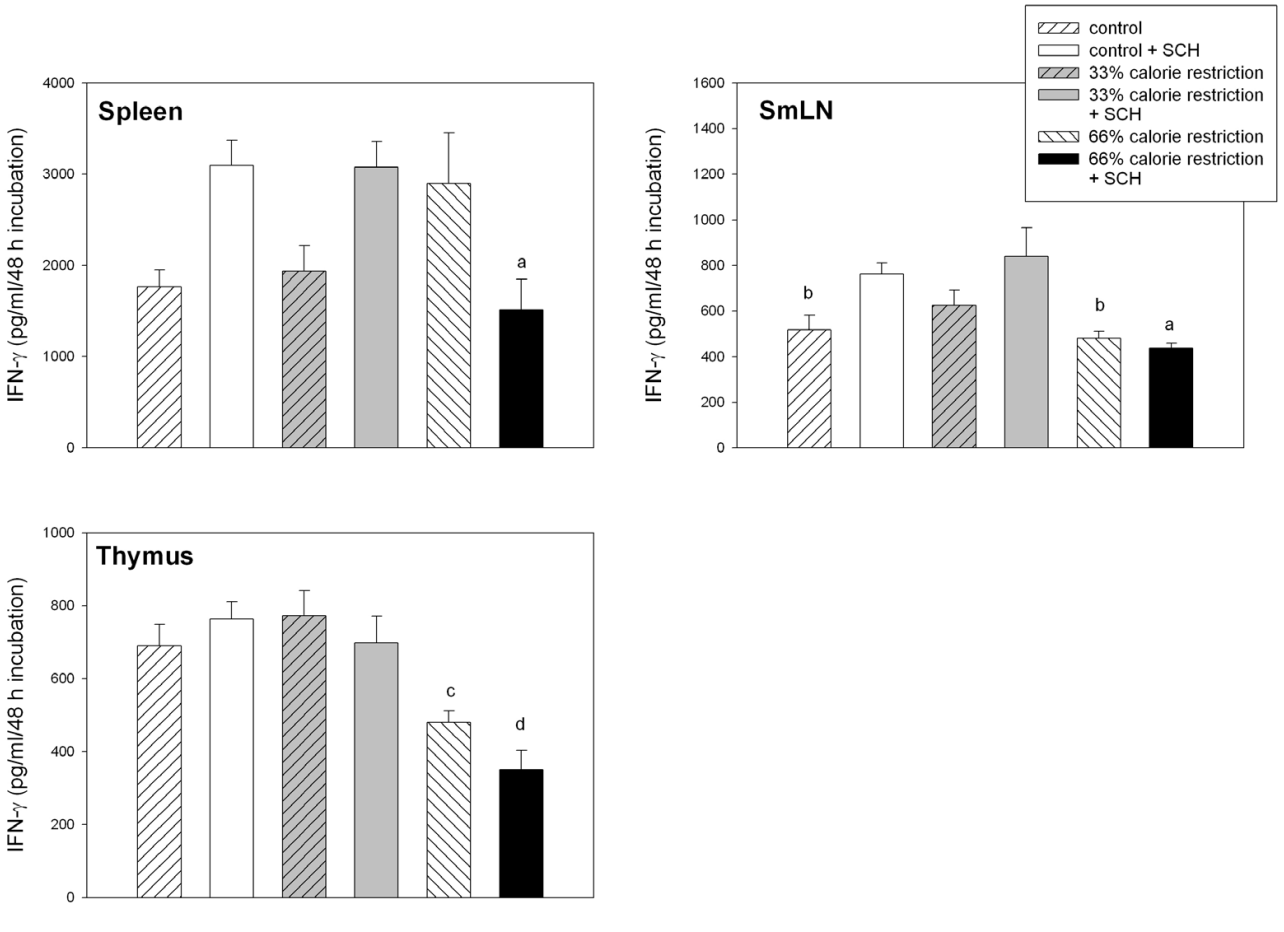
IFN-γ release from splenic, SmLN and thymic cells of control and calorie-restricted rats. Data shown as mean ± SEM (n = 7 rats/group). Letters indicate the existence of significant differences between groups after a one-way ANOVA followed by a Bonferroni's test, as follows: ^a ^p < 0.01 vs. control and 33 % calorie-restricted rats, both injected with SCH; ^b ^p < 0.02 vs. 33% calorie-restricted rats injected with SCH; ^c ^p < 0.02 vs. control rats injected with SCH; ^d ^p < 0.01 vs. control and 33% calorie-restricted rats regardless of SCH injection. For further statistical analysis, see text.

The changes in IFN-γ production by splenic, lymph node and thymic cells are depicted in Fig. 6. Factor analysis in the factorial ANOVA indicated the existence of significant interactions "diet x immunization" in the case of splenic and lymph node cells. In contrast to the increase in splenic IFN-γ production occurring in control and 33% calorie-restricted rats after SCH immunization there was a decrease in 66% calorie-restricted rats (F_2,36 _= 8.93, p = 0.0007). A similar trend was observed for lymph node IFN-γ production (F_2,36 _= 3.37, p < 0.0455). Thymic IFN-γ production was severely curtailed in 66% calorie-restricted rats (F_2,36 _= 13.5, p < 0.0001).

## Discussion

The foregoing results indicate that, in contrast to rats receiving a normal diet or a 33% calorie-restricted diet, rats subjected to severe (66%) calorie restriction do not exhibit clinical signs of EAE. The major immune findings in severely calorie-restricted rats were: (i) impaired splenic and lymph node mitogenic response to Con A and a higher splenic response to LPS; (ii) less splenic, lymph node and thymic CD4^+^, B and T-B cells, and splenic T cells; (iii) increased numbers of splenic and lymph node CD8^+ ^and CD4^+^- CD8^+ ^cells; (iv) impaired IFN-γ production in the three examined tissues.

Malnutrition produced by low or absent proteins in diet is linked to increased susceptibility to infection, often associated with severe marasmus or kwashiorkor. In contrast, calorie restriction of adult rodents by a diet enriched in proteins and low in fat and carbohydrates significantly increases immune responses [17-19]. Calorie restriction inhibits age-related dysregulation of cytokines and prevents, by enhancement of T cell apoptosis, accumulation of non-replicative, non-functional, senescent T cells [20]. The effect of calorie restriction is also demonstrable in young animals. For example, in young mice with experimental colitis, caloric restriction to 60 % of daily requirement augmented natural killer (NK) cell number and cytotoxicity and decreased IFN-γ release [21]. In a study using peripubertal male Wistar rats submitted to a 66 % calorie restriction diet similar to that employed herein for 4 weeks, we reported that calorie restriction modified 24 h rhythmicity of lymph node mitogenic responses to Con A and LPS, and of T, T-B, CD4^+ ^and CD4^+^-CD8^+ ^lymph node cell subsets. In addition, mean values of SmLN Con A response and CD4^+ ^cell number increased whereas those of B cell number and IFN-γ release decreased [22]. Collectively, the data are compatible with the view that T-cell responses increase in growing rats fed with a calorie-restricted diet enriched in proteins and low in fat and carbohydrates.

The proinflammatory cytokine IFN-γ, which is secreted by activated T lymphocytes and natural killer cells, stimulates the expression of MHC class I and II molecules on a wide variety of cells and is involved in the activation of macrophages and microglia [23]. Evidence suggests that IFN-γ plays a deleterious role in immune-mediated demyelinating disorders such as MS and EAE [24]. This cytokine, which is not normally present within the CNS, is detectable during the symptomatic phase of these disorders, and much of the pathology observed is consistent with IFN-γ involvement. Moreover, the treatment of MS patients with IFN-γ exacerbates the disease [25] and IFN-γ-secreting T cells can adoptively transfer EAE [26]. Transgenic mice that ectopically express IFN-γ in the CNS display a tremoring phenotype and myelin abnormalities [27] as well as increased susceptibility to EAE [28].

The foregoing results indicate the occurrence of a significant decrease in splenic, lymph node and thymus IFN-γ production in severely calorie-restricted rats, thus agreeing with observations indicating that, in autoimmune-prone mice, calorie restriction lowers mRNA expression of this cytokine [29], and that in young mice with experimental colitis, caloric restriction augments NK cytotoxicity and decreases IFN-γ levels [21].

It must be noted, however, that there is conflicting evidence as to whether IFN-γ provides a disease-reducing role in immune-mediated demyelinating disorders. The administration of antibodies to IFN-γ enhances the severity of EAE, whereas treatment of mice experiencing EAE with IFN-γ results in improved survival [30,31]. It has also been shown that mice with a mutation in either the gene encoding IFN-γ or its receptor are susceptible to EAE and that mouse strains that are normally resistant to EAE become susceptible when incapable of synthesizing or responding to this cytokine [32,33]. Clearly, the role that IFN-γ plays in demyelinating disorders is complex.

Summarizing, the present results are compatible with the view that the acute phase of EAE can be significantly curtailed by severe calorie restriction, presumably through an impaired IFN-γ production. The immunological status of rodents fed a calorie-restricted diet is superior to the immunological status of the non-restricted animals and through this mechanism caloric restriction may retard EAE development. Indeed, experimental calorie restriction (e.g. 25–50 % reduction of caloric intake), without deficiency in essential nutrients, may be a useful manipulation in slowing MS evolution [1].

However, caution should be taken in extrapolating the present experimental results to the clinical situation in view of the fact that monophasic EAE, particularly in the Lewis rat model, is a disease that shares only some aspects of human MS. In addition, EAE is defined in the present study on clinical grounds only and further histopathological and immunological examination of the brain is needed before a clear picture on what is happening in calorie restricted rats during EAE is obtained.

## Competing interests

The authors declare that they have no competing interests.

## Authors' contributions

AIE, PC, VJ-O and MPF-M carried out the experiments. DPC and AIE designed the experiments. Also, DPC performed the statistical analysis and drafted the manuscript. All authors read and approved the final manuscript.
